# A Novel Preoperative Prediction Model Based on Deep Learning to Predict Neoplasm T Staging and Grading in Patients with Upper Tract Urothelial Carcinoma

**DOI:** 10.3390/jcm11195815

**Published:** 2022-09-30

**Authors:** Yuhui He, Wenzhi Gao, Wenwei Ying, Ninghan Feng, Yang Wang, Peng Jiang, Yanqing Gong, Xuesong Li

**Affiliations:** 1 Department of Urology, Peking University First Hospital, Beijing 100034, China; 2 Department of Urology, The Third Hospital of Hebei Medical University, Shijiazhuang 050052, China; 3 Department of Urology, The Second People’s Hospital of Wuxi, Wuxi 214002, China

**Keywords:** upper tract urothelial carcinoma, deep learning, early diagnosis, neoplasm staging, neoplasm grading

## Abstract

*Objectives*: To create a novel preoperative prediction model based on a deep learning algorithm to predict neoplasm T staging and grading in patients with upper tract urothelial carcinoma (UTUC). *Methods*: We performed a retrospective cohort study of patients diagnosed with UTUC between 2001 and 2012 at our institution. Five deep learning algorithms (CGRU, BiGRU, CNN-BiGRU, CBiLSTM, and CNN-BiLSTM) were used to develop a preoperative prediction model for neoplasm T staging and grading. The Matthews correlation coefficient (MMC) and the receiver-operating characteristic curve with the area under the curve (AUC) were used to evaluate the performance of each prediction model. *Results*: The clinical data of a total of 884 patients with pathologically confirmed UTUC were collected. The T-staging prediction model based on CNN-BiGRU achieved the best performance, and the MMC and AUC were 0.598 (0.592–0.604) and 0.760 (0.755–0.765), respectively. The grading prediction model [1973 World Health Organization (WHO) grading system] based on CNN-BiGRU achieved the best performance, and the MMC and AUC were 0.612 (0.609–0.615) and 0.804 (0.801–0.807), respectively. The grading prediction model [2004 WHO grading system] based on BiGRU achieved the best performance, and the MMC and AUC were 0.621 (0.616–0.626) and 0.824 (0.819–0.829), respectively. *Conclusions*: We developed an accurate UTUC preoperative prediction model to predict neoplasm T staging and grading based on deep learning algorithms, which will help urologists to make appropriate treatment decisions in the early stage.

## 1. Introduction

Upper tract urothelial carcinoma (UTUC) is a relatively rare group of tumours, accounting for 5–10% of urothelial carcinomas [1]. Radical nephroureterectomy (RNU) is considered the standard of care for nonmetastatic UTUC, and should be accompanied by lymphatic dissection for patients with a locally progressive disease [2]. However, treatment strategies that preserve the kidney are reasonable for selected patients with low-stage UTUC, including endoscopic ablation and segmental ureterectomy. In low-risk patients, kidney preservation treatment is beneficial to protect renal function and avoid complications associated with radical surgery. There is no significant difference in 5-year cancer-specific survival after surgery versus RNU [3]. Neoadjuvant chemotherapy may be more beneficial for advanced UTUC because the loss of renal function after RNU may make the patient unsuitable for cisplatin application, which is currently one of the most effective chemotherapeutic agents in uroepithelial carcinoma [4]. The selection of appropriate patients is a significant challenge for urologists due to the limitations of imaging techniques and biopsy techniques [5].

If the pathologic characteristics of the tumour can be accurately predicted from routine clinical data before surgery, this information may improve the urologist’s strategy for the treatment of the disease. Predicting the stage and grade of the tumour may influence the choice of the first treatment for patients with UTUC and whether it should involve conservative treatment, RNU, RNU with lymph node dissection, or neoadjuvant systemic chemotherapy. The appropriate selection of patients for individualized treatment is beneficial in managing UTUC [6].

In recent years, prediction tools based on deep learning algorithms have developed rapidly, especially in the field of oncology. Although there have been some multivariate models based on preoperative information to predict the pathological features of postoperative UTUC [7,8,9], no relevant studies have applied deep learning algorithms to address this problem. Preliminary studies of deep learning prediction models have shown better performance than traditional multivariate prediction models [10,11,12]. As a national high-volume centre for UTUC in China [13], we are interested in determining how deep learning algorithms can identify the staging and grading of UTUC based on our relatively large sample size.

Therefore, the purpose of our study was to construct a preoperative prediction model for UTUC based on five deep learning algorithms to predict the staging and grading of UTUC to guide clinical decision-making.

## 2. Materials and Methods

**Figure 1:**
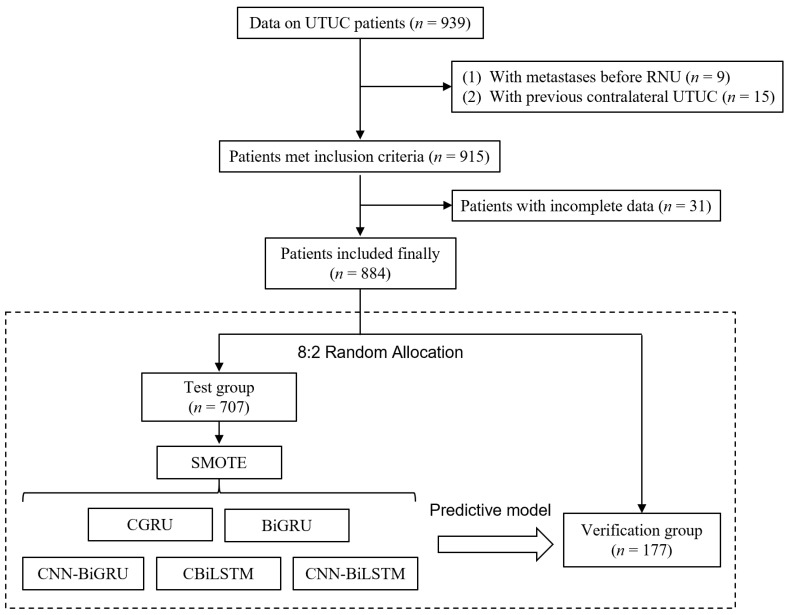
Flowchart of the study. UTUC, upper tract urothelial carcinoma; RNU, radical nephroureterectomy.

This analysis was reported according to the TRIPOD (transparent reporting of a multivariable prediction model for individual prognosis or diagnosis) guidelines, a reporting specification for predictive models of disease diagnosis and prognosis [14]. A flow diagram of the study is shown in Figure 1.

### 2.1. Patient Selection

We retrospectively collected the clinicopathological data of UTUC patients (884 cases) who underwent RNU surgery at Peking University First Hospital from 2001 to 2012. Patients in this study met the following inclusion criteria: (1) UTUC confirmed pathologically after surgery; (2) no distant metastasis. The following patients were excluded: (1) UTUC with metastases before RNU and (2) patients with previous contralateral UTUC. In addition, cases with incomplete data were excluded. Follow-up data were obtained by reviewing the clinical and pathological databases at our institution. Overall survival (OS) was calculated from the date of surgery to the date of all-cause death.

### 2.2. Feature Selection and Model Predictive Indicators

The information collected included medical record information and auxiliary test results. Preoperative information on UTUC patients included general information, past history, personal history, laboratory tests, and auxiliary examinations. Laboratory tests included haematology tests, coagulation tests, and biochemical examinations. Auxiliary examinations included, but were not limited to, the presence of hydronephrosis, tumour site, tumour location, and longest diameter of the tumour. Cases with incomplete data were excluded.

For feature selection, we first manually removed some features that were obviously irrelevant to the prediction results, such as the case ID. Subsequently, Xgboost (a machine-learning algorithm) was used to perform feature-correlation analysis to filter out features with low correlation to the prediction results, while the literature review was combined with the screening to retain the important features. The features were then modelled separately using deep learning models to compare their impact on the prediction results. By comparing and analysing different feature combinations, forty-four features were ultimately screened out (Supplementary Table S1).

The endpoint indicators predicted by the model were the specific staging and grading of UTUC. We treated the prediction results of the model as a discrete classification problem due to a lack of reliable weighting references for different T-stages or gradings and simplified the development of models. Information on the staging and grading of UTUC was obtained by the pathologic evaluation of postoperative samples. Clinical samples were obtained by experienced urologic oncologic surgeons using a standardized RNU approach, including resection of the full length of the kidney and ureter and the adjacent portion of the bladder cuff. All surgical samples were processed according to standard pathology procedures. Tumour staging was evaluated according to the 2002 Union for International Cancer Control (UICC) TNM classification of malignancies. There are two different clinical grading systems for UTUC at this time: the 1973 World Health Organization (WHO) classification and the 2004 WHO classification. No consensus has been made on which classification should supersede the other, and both are recommended in the European Association of Urology guidelines [15]. Tumour grading was assessed according to both the 1973 WHO classification and 2004 WHO classification in this study. Two specialist genitourinary pathologists independently reviewed each case. When a dispute arose, the decision was discussed with a third genitourinary pathologist. Three cases diagnosed as papillary urothelial neoplasms of low malignant potential according to the 2004 WHO classification were removed in the construction of the follow-up model because the number of cases was small.

### 2.3. Deep Learning and Model Construction

Data were randomly split into training and test sets at a ratio of 8:2 using random functions in Python. The training set was used to generate the prediction model, and the test set was used to estimate the model’s accuracy. To improve the ability to generalize and balance the different classes, the SMOTE algorithm was employed to counter the class imbalance. By combining the oversampling of the minority class and the undersampling of the majority class, SMOTE can achieve a better classifier performance [16].

We used five newer deep learning models that have been proposed in recent years in the biomedical field to predict neoplasm staging and grading in patients with UTUC, including CGRU, BiGRU, CNN-BiGRU, CBiLSTM, and CNN-BiLSTM.

CGRU is a multilabel classifier based on deep CNN. CNNs are a unique class of neural network models designed to identify hidden patterns and relationships in large datasets. GRU uses two gates: an update gate and a reset gate. The reset gate determines the amount of past information to be forgotten, while the update gate determines which information to keep and not to keep [17].

In the BiGRU network, the input vector (forward) and the corresponding reverse version (backwards) are fed into two GRUs, and the combination of the forward hidden-state output and the reverse hidden-state output is the output of the network [18].

CNN-BiGRU can automatically measure and assign weights to different leads based on their contributions. In short, the CNN module exploits interrelated features between leads and extracts differentiated spatial features. In addition, the BiGRU module extracts the underlying temporal features within each lead. The spatial and temporal features from these two modules are fused as global features for classification [19].

CBiLSTM is a two-channel hybrid neural network model based on CNN and BiLSTM. CNN and BiLSTM extract features from the original data and then connect them and map them to a fully connected layer. BiLSTM consists of two independent LSTM neural networks with a specific network structure consisting of an input gate structure and an output gate structure. The gates only restrict the direction of the information flow, and the LSTM affects the state of the RNN one at a time through the gate structure [20].

The CNN-BiLSTM neural network framework involves feature-extraction using a pretrained convolutional network and then feeds the feature vectors to a bidirectional long- and short-term memory network to capture the temporal features of the data [21]. The preceding CNN layers in the models can help first to extract abstract features and then provide them as inputs to the following RNN layers [22].

The hyperparameters were adjusted during model construction to construct high-quality models, such as the number of layers and hidden cells, learning rate, learning rate decay, dropout rate, batch size, and epoch.

### 2.4. Performance Verification

Considering the class imbalance that often occurs in biomedical datasets, we used the Matthews correlation coefficient (MCC), which is a more-reliable statistical rate in binary classification evaluation, to assess the model’s ability [23]. The receiver operating characteristic (ROC) curve with the area under the curve (AUC) and F1-scores, which are commonly used evaluation metrics, were also used to evaluate the performance of each predictive model. Internal validation was performed using 1000 bootstrap resamples.

### 2.5. Statistical Analysis

Continuous variables were expressed as the interquartile range. Pearson’s chi-squared test was conducted to analyse unordered categories data. Linear-by-linear association was used for ordinal data. A normality test was used for continuous data, and Student’s T test was used for data conforming to normal distribution. The log-rank test was used to compare the difference in survival curves between groups. Python 3.8.1 for Windows (https://www.python.org/, accessed on 14 July 2022) was used for deep learning analysis. Other analyses were performed with R statistical software version 3.4.1 (R Core Team, Vienna, Austria). *p* < 0.05 was considered as statistically significant.

## 3. Results

### 3.1. Patient Characteristics

**Figure 2:**
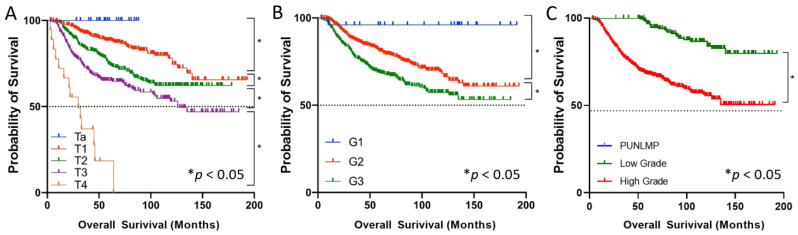
Overall survival curves based on different T-staging and grading in patients with UTUC. (**A**) Overall survival curves based on different T stages. (**B**) Overall survival curves based on the 1973 WHO grading classification. (**C**) Overall survival curves based on the 2004 WHO grading classification. * The overall survival time between the two groups was significantly different.

A total of 884 patients with UTUC were finally included in this study. The patients’ median (interquartile range interquartile range, IQR) age was 69 (61, 75) years old. Among the T-staging of tumours, Ta, T1, T2, T3, and T4 stage accounted for 2.7%, 34.2%, 33.8%, 27.1%, and 2%, respectively. Among the G stages of tumours, G1, G2, and G3 accounted for 2.8%, 56.1%, and 41.0%, respectively. The mean follow-up time of the patients was 70.3 months. The clinical and pathological characteristics of patients with UTUC are summarized in Table 1. Overall survival curves based on different stages and grades in patients with UTUC are shown in Figure 2. The overall survival of UTUC patients was significantly decreased with increased T staging and grading (*p* < 0.05).

### 3.2. Performance of Different Models

**Figure 3:**
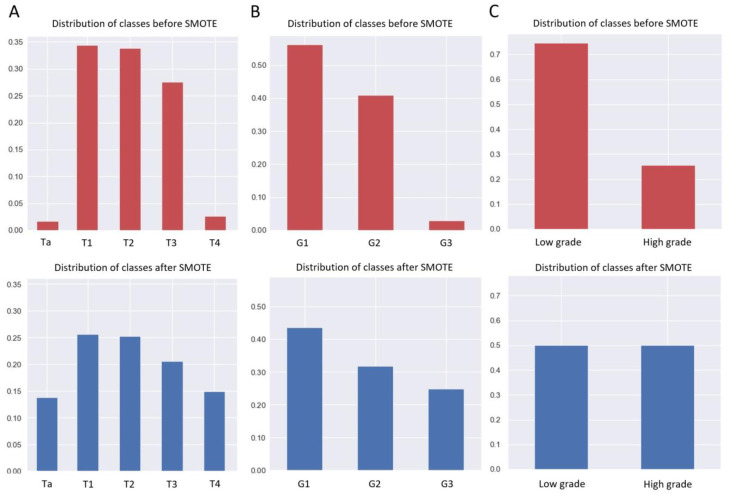
Distribution of classes before and after SMOTE in T staging and grading in the test set. (**A**) Distribution of classes before and after SMOTE in T staging. (**B**,**C**) Distribution of classes before and after SMOTE in 1973 World Health Organization (WHO) grading system and 2004 WHO grading system.

The full dataset was randomly divided into two exclusive datasets, with 80% being the training set (*n* = 707) and 20% the test set (*n* = 177). There were no statistically significant differences in the characteristics between the two sets (*p* > 0.05) (Supplementary Table S2). To solve the unbalanced problem of the test set data, we first used the SMOTE algorithm to balance the test set data. For the distribution of neoplasm T-staging and grading, the balance of the test set before and after using the SMOTE algorithm is shown in Figure 3.

Through multiple rounds of training and manual debugging, the final critical hyperparameters of the deep learning models were as follows: learning rate = 0.001, Adam = True, optim momentum value = 0.9, weight decay = 1 × 10^−8^, and batch size = 16.

**Figure 4:**
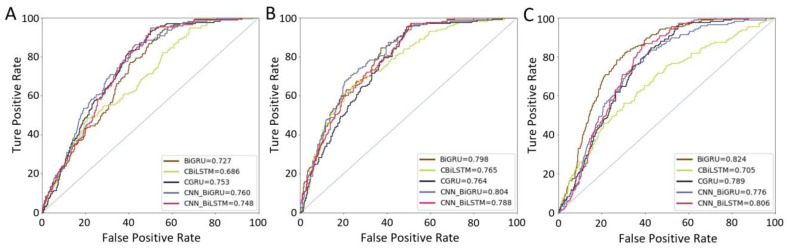
The ROC curve in the neoplasm T staging and grading of different deep learning prediction models. (**A**) The ROC curve in neoplasm T staging of different deep learning prediction models. (**B**) The ROC curve based on the 1973 WHO grading classification of different deep learning prediction models. (**C**) The ROC curve based on the 2004 WHO grading classification of different deep learning prediction models.

The ROC curves for the neoplasm T staging and grading of different deep learning models are shown in Figure 4. The T-staging prediction model based on CNN-BiGRU achieved the best performance, and the MMC, AUC, and F1 score were 0.598 (0.592–0.604), 0.760 (0.755–0.765), and 0.484 (0.479–0.489), respectively. The grading prediction model [1973 World Health Organization (WHO) grading system] based on CNN-BiGRU achieved the best performance, and the MMC, AUC, and F1 score were 0.612 (0.609–0.615), 0.804 (0.801–0.807), and 0.608 (0.605–0.611), respectively. The grading prediction model [2004 WHO grading system] based on BiGRU achieved the best performance, and the MMC, AUC, and F1 score were 0.621 (0.616–0.626), 0.824 (0.819–0.829), and 0.617 (0.612–0.622), respectively. Table 2 shows the performance of each model on the validation data.

## 4. Discussion

UTUC is a relatively rare tumour of the urinary tract for which RNU is the standard of treatment. For patients assessed as low-risk, nephron-sparing treatments such as endoscopic ablative treatments may be appropriate [24]. In the case of high-risk nonmetastatic UTUC patients, lymph node dissection or perioperative chemotherapy should be considered [25]. Hence, it is critical to evaluate the staging of the tumour accurately before deciding on treatment. The clinical stage of UTUC can be determined by ureteroscopy specimens combined with imaging, but the clinical and pathological stage of UTUC is usually discordant [26,27]. We, therefore, need more accurate predictive pathological tools to develop personalized treatment strategies for UTUC.

Our study used multiple deep learning algorithms to construct a preoperative prediction model for UTUC for the first time. It achieved AUCs of 0.760, 0.804, and 0.824 for tumour T staging (CNN-BiGRU), tumour grading based on the 1973 WHO classification (CNN-BiGRU), and tumour grading based on the 2004 WHO classification (BiGRU), respectively, demonstrating a better prediction performance. These results indicate that the deep learning model has a good application value as a preoperative prediction model in patients with UTUC. Previously, other investigators published tools of UTUC to predict muscle-invasive disease and/or nonorgan-confined disease. Table 3 summarizes the publications on preoperative prediction models for UTUC over the last ten years [7,8,9,13,28,29,30,31,32]. In contrast, our study has the following characteristics. First, previous studies used a prediction model based on multivariate analysis, which predicted a muscle-invasive disease or nonorgan-confined disease. Our study used a new deep learning algorithm developed to construct the prediction model in recent years. The feasibility and accuracy were confirmed in other studies [19,33,34,35] and achieved better prediction results. Compared with previous studies in which the predicted outcome was muscle-invasive disease or nonorgan-confined disease, our model predicts specific tumour T-staging and grading, with a richer clinical reference value. Based on the survival analysis of nearly 200 months in the overall cohort of UTUC patients in this study (Figure 2), we found significant differences in their overall survival prognosis in T2, T3, and T4 included in muscle-invasive disease and T3 and T4 included in nonorgan-confined disease. This suggests that we should pay more attention to personalized treatment selection in the above-categorized populations. For patients with predicted tumour stage T3, T4, or high tumour grade, more aggressive treatment strategies should be actively pursued to benefit patients, such as neoadjuvant systemic chemotherapy followed by surgery [36]. Conversely, patients with Ta stage and selective T1, who have a better prognosis, should be considered for kidney-preserving surgery to avoid postoperative renal loss and improve their quality of life. Moreover, although UTUC is a relatively rare tumour, as a national high-volume centre for UTUC in China, a relatively large number of cases were included in our study, and the cases were well represented [13]. A significant portion of the previous studies of preoperative dichotomous prediction models for UTUC did not perform model predictive validation, and our more-complex multivariate output model obtained better predictive performance in internal validation, suggesting the application prospects of deep learning algorithms in clinical prediction models.

Deep learning models have been widely used in many fields, such as environmental atmosphere prediction and financial-risk models. They have been rapidly developed in the medical field in the recent years [37]. Deep learning applications have been used successfully in cardiovascular, pulmonary, and urological diseases [38,39,40]. However, deep learning applications in UTUC are rare at the present stage. Lazo et al. proposed a spatial-temporal ensemble of convolutional neural networks for lumen segmentation to identify UTUC during ureteroscopy [41]. As an exploration of ureteroscopic image recognition, this technology is still far from clinical application. Deep learning methods can be further applied to various aspects of UTUC, such as tumour image diagnosis, prognosis analysis, and drug efficacy evaluation.

There are some potential limitations in our study. First, as a deep learning model, cases in the test set are still insufficient, and further samples need to be included in the future to improve the model’s performance. Second, it is necessary to validate this in a multicentre or international cohort model soon. In addition, since the deep learning model is a “black box” model and some computational principles are challenging to explain, we will consider developing a visualization program in the future to facilitate clinical promotion and application.

## 5. Conclusions

In contrast to the traditional multivariate model, we have developed an accurate UTUC preoperative prediction model to predict neoplasm T staging and grading based on deep learning algorithms, which will help urologists to make appropriate treatment decisions in the early stage.

## Figures and Tables

**Table 1:** **Clinical and pathological characteristics in patients with UTUC.** IQR, interquartile range; UBC, urothelial bladder carcinoma; PUNLMP, papillary urothelial neoplasm of low malignant potential.

Variables	No. Pts (%)
Total	884
Gender	
Male	395 (44.7)
Female	489 (55.3)
Age, median (IQR)	69 (61, 75)
BMI, kg/m^2^, median (IQR)	24.2 (22.0, 26.3)
History of UBC	
No	833 (94.2)
Yes	51 (5.8)
Smoking	
No	743 (84.0)
Yes	141 (16.0)
Hydronephrosis	
No	349 (39.5)
Yes	535 (60.5)
Tumour site	
Left	450 (50.9)
Right	434 (49.1)
Tumour location	
Renal pelvis	490 (55.4)
Ureter	394 (44.6)
Tumour diameter (cm), median (IQR)	3.0 (2.0, 4.2)
Pathological T stage	
Ta	24 (2.7)
T1	302 (34.2)
T2	299 (33.8)
T3	240 (27.1)
T4	19 (2.1)
WHO 1973 grade	
G1	25 (2.8)
G2	496 (56.1)
G3	362 (41.1)
WHO 2004 grade	
PUNLMP	3 (0.3)
Low grade	225 (25.5)
High grade	656 (74.2)
Overall survival	
Number	884
Mean follow-up times	70.3
Follow-up range	[3, 193]

**Table 2:** **Performance of each model on validation data.** MMC, Matthews correlation coefficient; AUC, area under the curve; WHO, World Health Organization. The 95% confidence intervals are shown in parentheses.

Models	T-Staging			Grading Based on the 1973 WHO Classification			Grading Based on the 2004 WHO Classification		
	MMC	AUC	F1 Score	MMC	AUC	F1 Score	MMC	AUC	F1 Score
BiGRU	0.532 (0.525–0.539)	0.727 (0.722–0.732)	0.410 (0.405–0.415)	0.604 (0.599–0.609)	0.798 (0.793–0.803)	0.625 (0.620–0.630)	0.621 (0.616–0.626)	0.824 (0.819–0.829)	0.617 (0.612–0.622)
CBiLSTM	0.482 (0.477–0.487)	0.686 (0.681–0.691)	0.371 (0.366–0.376)	0.566 (0.592–0.600)	0.765 (0.759–0.771)	0.576 (0.570–0.582)	0.511 (0.507–0.515)	0.705 (0.701–0.709)	0.396 (0.391–0.401)
CGRU	0.554 (0.549–0.559)	0.753 (0.747–0.759)	0.482 (0.476–0.488)	0.565 (0.558–0.572)	0.764 (0.758–0.770)	0.574 (0.568–0.580)	0.596 (0.590–0.602)	0.789 (0.783–0.795)	0.607 (0.601–0.613)
CNN-BiGRU	0.598 (0.592–0.604)	0.760 (0.755–0.765)	0.484 (0.479–0.489)	0.612 (0.609–0.615)	0.804 (0.801–0.807)	0.608 (0.605–0.611)	0.578 (0.574–0.582)	0.776 (0.772–0.780)	0.593 (0.589–0.597)
CNN-BiLSTM	0.542 (0.536–0.548)	0.748 (0.743–0.753)	0.451 (0.446–0.456)	0.595 (0.588–0.602)	0.788 (0.781–0.795)	0.602 (0.595–0.609)	0.615 (0.609–0.621)	0.806 (0.800–0.812)	0.605 (0.599–0.611)

**Table 3:** **Preoperative prediction tools in patients with upper tract urothelial carcinoma.** PPV, positive predictive value; NPV, negative predictive value; AUC, area under the curve; HGB, haemoglobin.

Author	Publication Years	Prediction Form	Outcome	No. of Patients	Variables	Evaluation Index	Validation
Brien et al. [8]	2010	Preoperative risk group stratification	Nonorgan-confined disease	172	Hydronephrosis, ureteroscopic grade, and urinary cytology	PPV 73% NPV 100%	None
Brien et al. [8]	2010	Preoperative risk group stratification	Muscle-invasive disease	172	Hydronephrosis, ureteroscopic grade, and urinary cytology	PPV 89% NPV 100%	None
Margulis et al. [9]	2010	Preoperative nomogram	Nonorgan-confined disease	659	Grade, architecture, and location	76.6% AUC	Internal
Favaretto et al. [7]	2012	Preoperative risk group stratification	Nonorgan-confined disease	274	Ureteroscopic grade, location, invasion, and hydronephrosis on imaging	70% AUC	None
Favaretto et al. [7]	2012	Preoperative risk group stratification	Muscle-invasive disease	274	Ureteroscopic grade, location, invasion, and hydronephrosis on imaging	71% AUC	None
Chen et al. [13]	2013	Preoperative nomogram	Nonorgan-confined disease	693	Gender, architecture, multifocality, location, and grade	79% C-index	Internal
Chen et al. [13]	2013	Preoperative nomogram	Muscle-invasive disease	693	Gender, architecture, multifocality, location, and grade	79% C-index	Internal
Jeon et al. [28]	2017	Preoperative nomogram	Nonorgan-confined disease or muscle-invasive disease	172	Urine cytology, hydronephrosis, local invasion, lamina propria invasion, high-grade tumour, and ureteroscopic scoring	82% AUC	None
Petros et al. [29]	2019	Preoperative nomogram	Nonorgan-confined disease	566	Clinical stage, biopsy tumour grade, tumour architecture, and HGB levels	82% C-index	Internal and external
Ma et al. [30].	2020	Preoperative nomogram	Muscle-invasive disease	245	Age, sessile, urine cytology, ureteroscopic, and high-grade biopsy	78% AUC	None
Yoshida et al. [31]	2020	Preoperative nomogram	Muscle-invasive disease	1101	Neutrophil to lymphocyte ratio, chronic kidney disease, local invasion on imaging, tumour location, and hydronephrosis	77% AUC	Internal and external
Wang et al. [32]	2021	Preoperative nomogram	Muscle-invasive disease	4149	Age, tumour size, T-stage, N-stage, M-stage, LN surgery, histology, radiation, and chemotherapy	74% C-index	Internal and external

## Data Availability

The original contributions presented in the study are included in the article/Supplementary Materials. Further inquiries can be directed to the corresponding authors.

## Supplementary Materials

The following supporting information can be downloaded at: https://www.mdpi.com/article/10.3390/jcm11195815/s1, Table S1: Features selection to construct models. Table S2: Comparison of basic characteristics between training set and test set.
