# Supplementary material for: A Novel Preoperative Prediction Model Based on Deep Learning to Predict Neoplasm T Staging and Grading in Patients with Upper Tract Urothelial Carcinoma
Source: J Clin Med. 2022 Sep 30;11(19):5815. doi: 10.3390/jcm11195815 (PMC9571440; doi:10.3390/jcm11195815)
Supplement: Supplementary file 1 [file jcm-11-05815-s001.zip › jcm-1843301-supplementary.pdf]

Supplementary Materials

Table S1. Features selection to construct models. BMI, body mass index; UBC, urothelial bladder carcinoma; AA, aristolochic acid; CKD, chronic kidney disease; CT, computed tomography.

Features for Model's Construction Base on Preoperative Information	
General information	Sex, Age, BMI
Past history	History of UBC, History of AA medication, History of nonurinary tumour, Diabetes, Hypertension, Coronary artery disease
Personal history	Smoking, Drinking
Hematology tests	White blood cells, Red blood cells, Platelet, Neutrophils, Lymphocytes, Neutrophils-lymphocytes ratio, Eosinophils, Basophils, ABO blood type, Rh blood type
Coagulation tests	Prothrombin Time, Activated partial thromboplastin time
Biochemical examination	Alanine aminotransferase, Aspartate aminotransferase, Total protein, Albumin, Prealbumin, Creatinine, Glomerular filtration rate, Stage of CKD, Sodium, Globulin, Triglycerides, Total cholesterol, High-density lipoprotein, Low-density lipoprotein,
Auxiliary examinations	Cystoscopy (positive or negative), Ureteroscopy (positive or negative), Urine cytology (international standard of PAP classification), Tumour site (left or right), Tumour location (renal pelvis or ureter), Maximum Tumour diameter (Measurement by CT images), Hydronephrosis (positive or negative)

Table S2. Comparison of basic characteristics between training set and test set. BMI, body mass index; IQR, interquartile range. UBC, urothelial bladder carcinoma; PUNLMP, papillary urothelial neoplasm of low malignant potential.

Variables	Training set	Test set	χ^2/T	*P*
	No. Pts (%)	No. Pts (%)		
Total	707	177		
Gender				
Male	313 (44.3)	82 (46.3)	0.242	0.623
Female	394 (55.7)	95 (53.7)		
Age, median (IQR)	69 (74,69)	70 (62,75)	1.430	0.153
BMI, kg.m^2, median (IQR)	23.9 (21.9, 26.2)	23.6 (20.6, 26.2)	−1.815	0.070
Previous bladder tumour				
No	666 (94.2)	167 (94.4)	0.006	0.939
Yes	41 (5.8)	10 (5.6)		
Smoking				
No	599 (84.7)	144 (81.4)	1.198	0.274
Yes	108 (15.3)	33 (18.6)		
Hydronephrosis				
No	287 (40.6)	62 (35.0)	1.835	0.175
Yes	420 (59.4)	115 (65.0)		
Tumour site				
Left	356 (50.4)	94 (53.1)	0.430	0.512
Right	351 (49.6)	83 (46.9)		
Tumour location				

Renal pelvis	399 (56.4)	91 (51.4)	1.446	0.229
Ureter	308 (43.6)	86 (48.6)		
Tumour diameter (cm), median (IQR)	3.1 (2.1, 4.5)	3.3 (2.0, 5.1)	1.648	0.100
Pathological T stage				
Ta	19 (2.7)	5 (2.8)		
T1	244 (34.5)	58 (32.8)		
T2	235 (33.2)	64 (36.2)	0.002	0.969
T3	194 (27.4)	46 (26.0)		
T4	15 (2.1)	4 (2.2)		
WHO 1973 grade				
G1	19 (2.6)	6 (3.4)		
G2	394 (55.7)	102 (57.6)	0.527	0.468
G3	294 (41.6)	69 (39.0)		
WHO 2004 grade				
PUNLMP	2 (0.3)	1 (0.6)		
Low grade	171 (24.2)	54 (30.5)	3.355	0.067
High grade	534 (75.5)	122 (68.9)		
